# Expanding Arsenal against Neurodegenerative Diseases Using Quercetin Based Nanoformulations: Breakthroughs and Bottlenecks

**DOI:** 10.2174/1570159X20666220810105421

**Published:** 2023-05-18

**Authors:** Sukriti Vishwas, Rajesh Kumar, Rubiya Khursheed, Arya Kadukkattil Ramanunny, Rajan Kumar, Ankit Awasthi, Leander Corrie, Omji Porwal, Mohammed F. Arshad, Mohammed Kanan Alshammari, Abdulrahman A. Alghitran, Ashwaq N. Qumayri, Saif M. Alkhaldi, Abdulaziz Khalaf Alshammari, Dinesh Kumar Chellappan, Gaurav Gupta, Trudi Collet, Jon Adams, Kamal Dua, Monica Gulati, Sachin Kumar Singh

**Affiliations:** 1 School of Pharmaceutical Sciences, Lovely Professional University, Phagwara 144411 Punjab, India;; 2 Department of Pharmacognosy, Faculty of Pharmacy, Tishk International University, Erbil, 44001, KRG, Iraq;; 3 Department of Research and Scientific Communications, Isthmus Research and Publishing House, New Delhi, 110044, India;; 4 Department of Pharmaceutical Care, Rafha Central Hospital, Rafha 91911, Saudi Arabia;; 5 Department of Clinical Pharmacy, General Administration of Pharmaceutical Care, Ministry of Health, Riyadh 11176, Saudi Arabia;; 6 Department of Pharmaceutical Care, King Khalid Hospital in Majmaah, Riyadh Region 76312, Saudi Arabia;; 7 College of Pharmacy, Northern Border University, Rafha 91911, Saudi Arabia;; 8 Department of Life Sciences, School of Pharmacy, International Medical University, Bukit Jalil, 57000, Kuala Lumpur, Malaysia;; 9 School of Pharmacy, Suresh Gyan Vihar University, Mahal Road, Jagatpura, Jaipur, India;; 10 Department of Pharmacology, Saveetha Dental College and Hospitals, Saveetha Institute of Medical and Technical Sciences, Saveetha University, Chennai, India;; 11 Innovative Medicines Group, Faculty of Health, Queensland University of Technology (QUT), Kelvin Grove, Brisbane, Queensland, 4059, Australia;; 12 Faculty of Health, Australian Research Centre in Complementary and Integrative Medicine, University of Technology Sydney, Ultimo, NSW, 2007, Australia;; 13 Discipline of Pharmacy, Graduate School of Health, University of Technology Sydney, Ultimo, NSW, 2007, Australia;; 14 Uttaranchal Institute of Pharmaceutical Sciences, Uttaranchal University, Dehradun, India

**Keywords:** Quercetin, antioxidant, neuroinflammation, neurodegenerative disease, novel drug delivery systems, Alzheimer’s disease

## Abstract

Quercetin (Qu), a dietary flavonoid, is obtained from many fruits and vegetables such as coriander, broccoli, capers, asparagus, onion, figs, radish leaves, cranberry, walnuts, and citrus fruits. It has proven its role as a nutraceutical owing to numerous pharmacological effects against various diseases in preclinical studies. Despite these facts, Qu and its nanoparticles are less explored in clinical research as a nutraceutical. The present review covers various neuroprotective actions of Qu against various neurodegenerative diseases (NDs) such as Alzheimer’s, Parkinson’s, Huntington’s, and Amyotrophic lateral sclerosis. A literature search was conducted to systematically review the various mechanistic pathways through which Qu elicits its neuroprotective actions and the challenges associated with raw Qu that compromise therapeutic efficacy. The nanoformulations developed to enhance Qu’s therapeutic efficacy are also covered. Various ongoing/completed clinical trials related to Qu in treating various diseases, including NDs, are also tabulated. Despite these many successes, the exploration of research on Qu-loaded nanoformulations is limited mostly to preclinical studies, probably due to poor drug loading and stability of the formulation, time-consuming steps involved in the formulation, and their poor scale-up capacity. Hence, future efforts are required in this area to reach Qu nanoformulations to the clinical level.

## 1 INTRODUCTION

Neurodegenerative diseases (NDs) are a group of conditions defined by gradual loss of neuronal function and accumulation of proteins with altered physicochemical characteristics in the central and peripheral nervous system [1]. In addition, genetic influences, oxidative stress [2], neuroinflammation [3], and abnormalities in mitochondrial functions can also lead to NDs. These NDs can affect the body's sensory and motor neurons markedly, causing cognitive, behavioural and psychotic abnormalities that affect the lifestyle with increased mental stress [4].

Quercetin (Qu), a dietary flavonoid, is obtained from many fruits and vegetables such as coriander, broccoli, capers, asparagus, onion, figs, radish leaves, cranberry, walnuts, and citrus fruits [5]. It is a secondary polyphenolic metabolite. It has a benzo-(γ)-pyrone skeletal structure with a carbon framework of C6-C3-C6, consisting of two benzene rings, A and B, connected by a three-carbon pyrone ring C [6]. Chemically, it is a pentahydroxyflavone since it has five hydroxyl groups at carbons 3,3’,4’,5, and 7 positions on its flavonol skeleton [7, 8]. Its glycosidic derivatives such as isoquercetin [9], hyperoside [10], quercitrin [11], and rutin [12] are also reported for many pharmacological activities [13]. The antioxidant and anti-inflammatory potential of Qu helps treat diseases like diabetes [14], hypertension [15], arrhythmia [16], atherosclerosis [17], renal dysfunctions, and various types of cancer such as lung, pancreatic, prostate, kidney, and breast [18-20]. The potential of Qu in treating different NDs has been proven through several clinical and preclinical trials [21].

The neuroprotective activity of Qu is attributed mainly to the inhibition of polyglutamine aggregation, acetylcholinesterase (AChE), amyloid-β fibrillogenesis, 6-hydroxydo-pamine, 3-nitropropionic acid, and enhanced in Apolipoprotein E (ApoE) level [22, 23]. However, Qu has been reported to possess low aqueous solubility and extensive first-pass metabolism that limits its oral bioavailability [24]. To date, several studies have been carried out to deliver Qu through a novel drug delivery system (NDDS) to enhance its oral bioavailability and target it to a specific site. The present review entails the mechanistic pathways involved in the pathogenesis of different NDs such as Alzheimer's disease (AD), Parkinson's disease (PD), Huntington's disease (HD), and Amyotrophic lateral sclerosis (ALS), as well as the mechanism through which Qu shows its protective action under these conditions. A comprehensive and critical review of various NDDS such as nanoemulsions (NEs) [25, 26], self-nanoemulsifying drug delivery system (SNEDDS) [27, 28], nanoliposome, solid lipid nanoparticles (SLNs) [29], nanostructured lipid carriers (NLCs), polymeric nanoparticles [30, 31], and metallic nanoparticles [32] to treat NDs that have been explored till date to enhance the therapeutic potential of Qu.

### 1.1 Global prevalence of NDs and economic burden

Globally, more than 44 million people have been diagnosed with dementia by 2021 [33]. Among them, about 70% of cases are due to AD [34]. More than 5.8 million people aged 65 years and above have been affected by AD in the USA till 2021. It is expected that the number may increase to 13.8 million by 2050 in the USA. The overall cost of health care, long-term care, and hospice services for people aged 65 and more with dementia was about $305 billion in 2020 [35]. In Europe, nearly 10.5 million people have been reported to suffer from dementia: among them, AD accounts for 60 to 80 percent of the cases. This figure is projected to become nearly double over the next 35 years and is expected to hit over 18 million by 2050 [36]. About 4.72 million Australians were diagnosed with dementia by the end of 2020, and this number is predicted to rise to 5.9 million by 2028 and 10.76 million by 2058 [34]. Similarly, in Japan, about 3.5 million people were suffering from AD till 2016, and data is expected to reach up to 4.6 million by 2026 [37]. In China, the prevalence rate of dementia was 5.60% till 2019 [38]. Currently, in India, nearly 4 million people are suffering from AD [33, 39].

PD disease affects more than ten million people globally. It has affected around one million people in the United States, which is higher than the total number of persons diagnosed with multiple sclerosis, muscular dystrophy, and Lou Gehrig's disease (or ALS). By 2030, this number is predicted to reach 1.2 million. Every year, over 0.06 million Americans are diagnosed with PD [40]. PD has a total direct and indirect cost of over $52 billion each year in the United States alone, including treatment, social security payments, and lost income [40]. On average, the medication cost for the treatment of PD is about $2,500 per year, but its surgical procedure/treatment might cost up to $0.1 million per individual [40]. In India, a total of 18,896 individuals were reported with PD till 2020 [41]. Overall, the gross prevalence of PD was 42.3 people per 0.1 million, but the prevalence among those over the age of 60 was 308.9 per 0.1 million, indicating an age-related increase in PD’s prevalence [41].

HD is another type of ND. Globally, nearly 5 to 8 persons in a population of 0.1 million were diagnosed with HD by 2021 [42, 43]. About 0.03 million people in the United States have been diagnosed with HD, and another 0.2 million are at risk of developing the condition. Symptoms commonly develop between a group of age 30 to 50 [44]. In India, around three to five individuals per 0.1 million people, or nearly 0.04-0.07 million of the total population (1397 million), have been estimated to have HD [44]. In Europe, around 3 to 7 persons per 0.1 million people are reported to have HD [44]. In South Africa, 46 persons per 0.1 million people have been reported to have HD. In the majority of European nations, the prevalence varies between 1.63 to 9.95 persons per 0.1 million persons. In Finland and Japan, the prevalence of HD is less than 1 per 0.1 million persons [44].

Another type of ND is ALS. Globally, ALS affects nearly one in every 0.05 million persons, resulting in 5,760 to 6,400 newly diagnosed cases each year. People are usually diagnosed with ALS in the age group of 50 to70. The majority of ALS instances are thought to be sporadic, with just around 8% to 10% of cases being inherited [45].

## 2 VARIOUS MOLECULAR MECHANISMS INVOL-VED IN NDS

### 2.1 Oxidative Stress

**Fig. (1):**
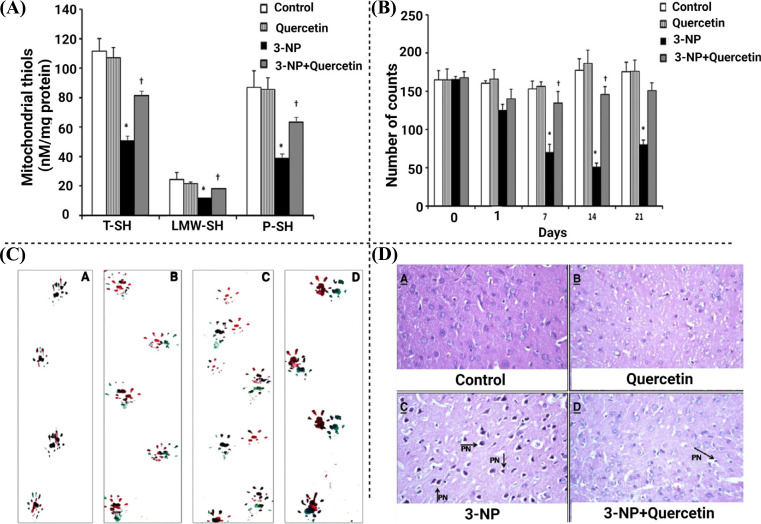
Effects of Qu on the treatment of HD [77].

Oxidative stress is a state in which an abnormal level of free radicals and antioxidants are reported in the body that is responsible for enhancing the production of reactive oxygen species (ROS) such as hydrogen peroxide (H_2_O_2_), nitric oxide, superoxide (O_2_•), highly reactive hydroxide radicals (OH¯), hydroxyl radicals (OH•). They originate from a range of sources such as aerobic systems, oxidative metabolism, nicotinamide adenine dinucleotide (NADPH) oxidase activity, and environmental stress such as ultraviolet (UV) radiation and contaminants which affect the neurons [46]. High oxygen consumption reduces the level of the antioxidant and regenerative capacity of the neuronal cells. Moreover, oxidative stress leads to cellular dysfunction and the generation of toxic species such as aldehydes, peroxides, ketones, and cholesterol oxide. Amyloid β (Aβ) intracellular and extracellular hydrogen peroxide (H_2_O_2_), calcium dysfunction, α-synucleins, copper and zinc-containing superoxide dismutase (Cu/ZnSOD), enhanced ROS and oxidative stress (oxidation of lipid, protein, and DNA) are responsible for oxidative damage which further leads to various NDs such as AD, PD, HD, and ALS [2]. In addition to oxidative stress, some other factors also cause neuroinflammation. They are discussed in subsequent sections (Fig. **1**).

### 2.2 Neuroinflammation

Neuroinflammation is one of the types of inflammation occurring in the central nervous system (CNS). Various factors that affect neuroinflammation are cytokines, chemokines, ROS, reactive nitrogen species (RNS), and secondary messengers. Various pro-inflammatory cytokines that contribute to neuroinflammatory responses include interleukin (IL)-1, IL-6, and tumor necrosis factor α (TNFα), and chemokines include chemokine (C-C motif) ligand (CCL)2, CCL5, and chemokine (C-X-C motif) ligand 1 (CXCL1). These are responsible for the production of resident CNS glia (microglia and astrocytes), endothelial cells, and peripherally derived immune cells. Neuroinflammation affects the general physiology of the brain and causes serious dysfunction such as edema, neuronal cells, tissue damage, immune cell recruitment, potential cell apoptosis as well as necrosis. These mediators are synthesized by activated resident CNS cells, such as microglia and astrocytes [47]. Nuclear factor-kappa, a light chain enhancer of activated B cells (NF-κB) signaling pathway, gets activated by TNFα and IL-6, IL-8 and IL-1β in microphase and leads to neuroinflammation as well as cell apoptosis [48].

### 2.3 Mitochondrial Dysfunction

Mitochondrion is a double membrane-bound organelle present in eukaryotic cells. It supplies energy demand in the form of ATP through the oxidative phosphorylation process [49]. Fusion, as well as fission proteins present in mitochondria, is mainly accountable for mitochondrial functioning. Mitochondrial dysfunction arises in NDs due to the accumulation of Aβ, formation of a neurofibrillary tangle, genes responsible for PD, mutation of Huntingtin (Htt) protein, and oxidative stress. In the case of AD, accumulation of Aβ causes inhibition of phosphorylation in mitochondria, whereas tau protein inhibits complex-1 activity, which is the key component of the electron transport chain [50, 51]. Overall, they lead to mitochondrial DNA damage. In the case of PD, accumulation of PARK7 (encoding DJ-1), α-synuclein, parkin, PINK1, or LRRK2 genes takes place [52]. Aggregation of these genes leads to the postponement of fusion of phagosomes with lysosomes during the mitophagy process, which further leads to increased oxidative stress in mitochondria. In the case of HD, the abnormal function of the Huntingtin (Htt) gene is responsible for the numerous dysfunctions of the cells. In mitochondria, mutated Htt genes restrict mitochondrial fission-fusion protein, which further leads to mitochondrial dysfunctions. These genes also directly interact with the mitochondrial membrane and enhance calcium sensitivity [53, 54].

## 3 PROTECTIVE ROLE OF QU IN NDS

### 3.1 Alzheimer’s Disease (AD)

Among the NDs, AD is the most common cause of dementia. The main symptom of this disease is cognitive dysfunction. It is generally seen in the geriatric population (more than 65 years of age) [35]. Several studies have revealed that degeneration of cholinergic neurons in the hippocampus and cerebral cortex of the brain may reduce cognitive functions and judgment capacity. Pathogenesis associated with the disease includes an accumulation of amyloid β and the formation of neurofibrillary tangle (NFT) in the cerebral cortex and hippocampus part of the brain. The formation of NFT has been observed during microscopic observation in post-mortem reports of the brain that belonged to patients having AD [55].

**Fig. (2):**
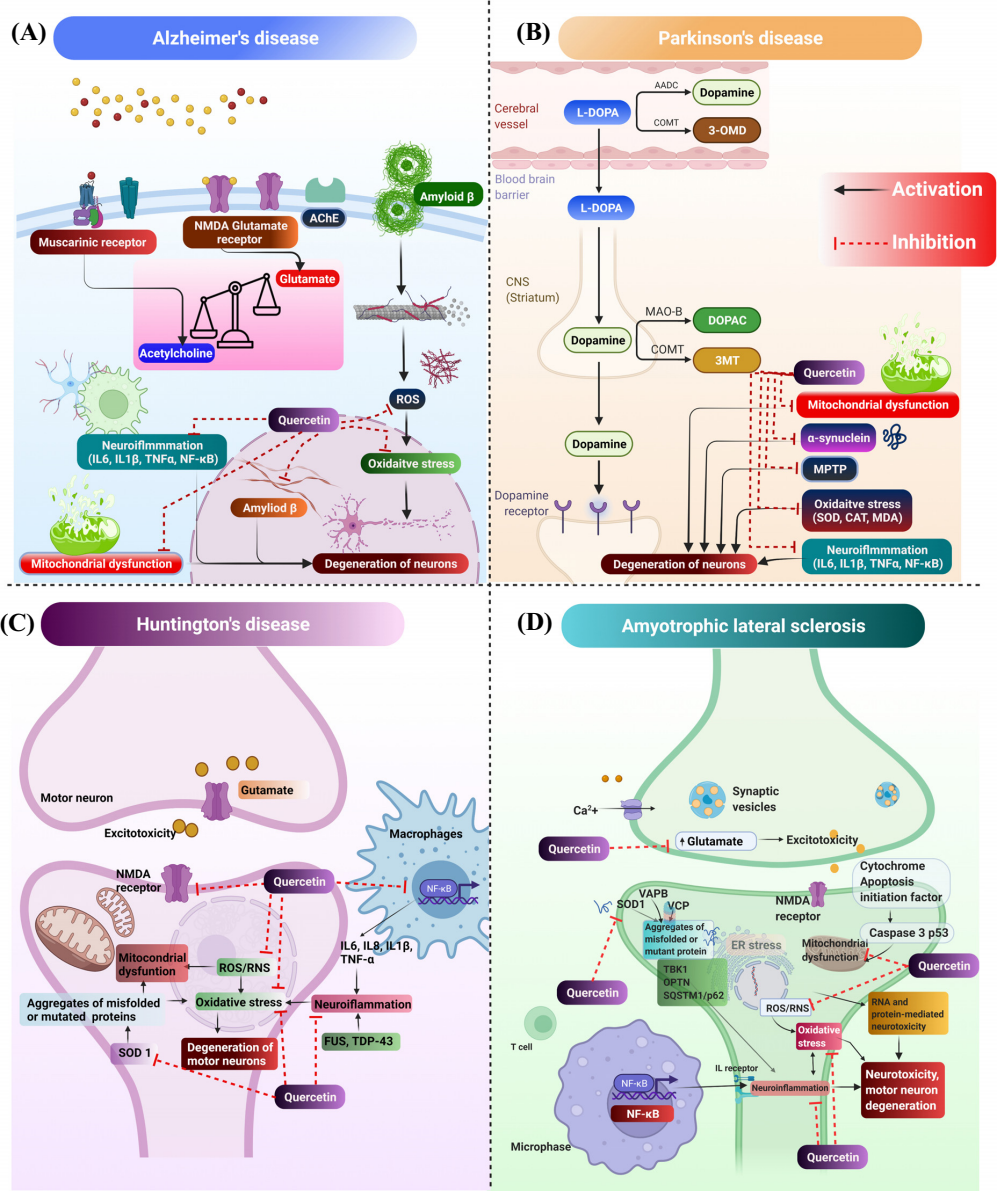
Mechanistic pathways deciphering the effect of Qu against (**A**) Alzheimer’s disease (AD), (**B**) Parkinson’s disease (PD), (**C**) Huntington’s disease (HD), and (**D**) Amyotrophic lateral sclerosis (ALS).

Several synthetic drugs have been approved for the treatment of AD, such as rivastigmine, galantamine, donepezil, and memantine [56] (Fig. **2**). These drugs work by inhibiting AChE and inhibition of NMDA glutamate receptors. Also, many preclinical and clinical studies have been reported where herbal and synthetic drugs have shown good effects for the treatment of AD [57]. In addition, some of the isolated phytoconstituents, such as lycopene, rutin, *etc.*, have shown good efficacy in treating AD. Qu is one of the flavonoids that has shown anti-AD effects by attenuating tau aggregation, accumulation of Aβ, deoxyribonucleic acid (DNA) damage, mitochondrial dysfunctions, and cognitive dysfunction [58]. A pictorial representation of the anti-AD effects of Qu is shown in Fig. (**2A**). Qu has been reported to possess strong antioxidant and anti-inflammatory properties. It inhibits the activation and production of ROS and RNS, leading to the reduction of oxidative stress. Qu also inhibits mitochondrial dysfunction and produces neuroprotective effects in various neurological diseases [58, 59].

Qu attenuates the level of ROS that further enhances Aβ aggregation/accumulation and Aβ induced neurodegeneration [60]. In previous studies, Qu has been reported to produce inhibitory effects against the AChE enzyme and reduce the metabolism of ACh in synapse as well as in pre-synaptic receptors, thereby enhancing cognitive functions [58, 61, 62]. Additionally, Aβ accumulation may increase calcium homeostasis, which leads to mitochondrial dysfunction and neuronal cell apoptosis [5]. Godoy *et al.* reported that Qu had shown antioxidant effects by inhibiting H_2_O_2_-induced neuronal toxicity. It has also been reported to reduce Aβ induced neurodegeneration and mitochondrial injury. Authors also reported that Qu reduced ROS formation [63]. Chen and co-workers studied the effects of Qu, which attenuated H_2_O_2_ and chemical anoxia-induced cell apoptosis. In addition, Qu inhibited ROS and showed positive effects against oxidative stress in rat glioma C6 cells [64]. According to Mehta *et al.,* Qu at a dose of 30 mg/kg has been reported to reduce chronic unpredicted stress (CUS) such as anxiety and depression and improve cognitive ability. The mice behaviour was also studied (plus maze and open field) for analyzing CUS. CUS is reported to enhance the level of oxidative stress markers (thiobarbituric acid reactive substances and nitric oxide) and pro-inflammatory cytokines (IL-6, TNF-α, Interleukin 1 beta, and Cyclooxygenase-2). This further leads to the apoptosis of neuronal cells in the hippocampus. The levels of all these inflammatory markers have been significantly reduced by Qu, and thus, neuronal cell damage was prevented [65]. Unsal *et al.* reported the effects of Qu on neuronal injury induced by cadmium in the frontal cortex in Sprague Dawley (SD) rats [66]. Qu showed antioxidant and anti-inflammatory actions and thereby produced neuroprotective effects. To evaluate these activities, various biochemical studies like malondialdehyde (MDA), superoxide dismutase (SOD), glutathione peroxidase (GPX), catalase (CAT), and histopathological examinations were performed. The results indicated that Qu showed antioxidant activities by attenuating the level of SOD, SPX, and CAT in the frontal cortex part of the rats and ameliorated the level of MDA. In addition, histopathological studies revealed that Qu reduced degeneration of the neuronal cell in the frontal cortex part of the brain [66]. In another study, Sabogal-Guáqueta *et al.* investigated the ability of Qu to attenuate amyloidosis, astrogliosis, and microgliosis in the hippocampus and amygdala by lowering Aβ (1-40) and Aβ (1-42) levels in an elderly triple-transgenic AD mice model. Simultaneously, an enhancement in cognitive and psychological function was also observed in mice model after administration of Qu [67].

### 3.2 Parkinson’s Disease (PD)

PD is an extrapyramidal motor disturbance that shows symptoms including rigidity, tremor, bradykinesia, mass-like face, *etc.* Pathogenesis of PD includes degeneration of dopaminergic neurons in substantial nigra and pars compacta part of the brain [68]. Moreover, the accumulation of α-synuclein protein has also been observed in PD [69]. Qu has been reported to have numerous neuroprotective effects through uplifting the level of dopamine in the extrapyramidal system of the brain and attenuating the accumulated levels of α-synuclein, 1-methyl-4-phenyl-1,2,3,6-tetrahydropyridine (MPTP), oxidative stress, neuroinflammation and apoptosis [70]. Singh and co-workers reported the combination effects of Qu and piperine against MPTP-induced PD in rats [71, 72]. The authors evaluated various parameters such as body weight, locomotor activity, oxidative stress, neuroinflammation, and altered neurotransmission of dopaminergic neurons. The results indicated that a combination of Qu (25 mg/kg) and piperine (2.5 mg/kg) significantly (*p*<0.05) restored rat’s body weight, locomotor activities, motor coordination, and grip strength induced by MPTP (100 μg/μL, intranigrally) in comparison to both the individual doses of Qu (25 and 50 mg/kg) when administrated alone. The combination of Qu and piperine also attenuated inflammatory cytokinins such as IL1β, IL6, and TNFα. This was due to an improvement in the oral bioavailability of Qu due to the presence of piperine [71]. In another study, Sharma and co-workers reported neuroprotective effects of Qu against aluminium-induced neurotoxicity as well as apoptosis in the hippocampus part of the brain in Wistar rats. Qu at a dose of 10 mg/kg/day reduced aluminium-induced oxidative stress as evident from the reduction in ROS level and enhanced level of mitochondrial superoxide dismutase activity. Moreover, Qu attenuated aluminium-induced translocation of cyt-c, up-regulation of Bcl-2, downregulation of Bax, p53, caspase-3 activation as well as reduced DNA fragmentation [73]. A pictorial representation of the anti-PD effects of Qu is presented in Fig. (**2B**).

### 3.3 Huntington’s Disease (HD)

HD is an orphan disease that involves an imbalance of neurotransmitters such as gamma-aminobutyric acid (GABA), glutamate, Ach, and dopamine. Common symptoms of HD include cognitive dysfunctions (dementia), motor dysfunction (chorea), and behavioural changes [74-76].

Qu is reported for the treatment of HD. In one of the studies, Sandhir *et al.* reported the effects of Qu on mitochondrial dysfunction induced by 3-nitropropionic acid (3-NP) for the treatment of HD in rats. The neurotoxic effects produced by 3-NP in the brain include oxidative stress, neuroinflammation, mitochondrial dysfunction, and poor motor control. The study was focused on mitochondrial swelling, mitochondrial bioenergetics, neurobehavioral deficits, oxidative stress, and histopathological changes. The results indicated that Qu reduced mitochondrial swelling and mitochondrial oxidative stress by minimizing the reaction cascade in the respiratory chain and restoring the levels of ATP (Figs. **1A**, **B**). The oral administration of Qu showed antioxidant effects by restoring catalase and superoxide dismutase (SOD) activity. Moreover, oral administration of Qu enhanced motor dysfunction, which was analyzed by narrow beam walking in footprints analysis (Fig. **1C**). The results of histopathological studies have shown enhanced irregular damaged cells with condensed and pyknotic nuclei in the striatum in 3-NP induced groups. Qu reverted the neurodegenerative changes induced by 3-NP (Fig. **1D**) [77]. In another study, Kuhad and co-workers reported the effects of Qu against quinolinic acid (QA) induced neurotoxicity in rats. QA enhanced neuroinflammation by increasing TNFα and reducing the level of neurotransmitters such as dopamine, serotonin, and norepinephrine in the forebrain part of the rat brain. Qu significantly attenuated these behavioural, biochemical, and neurochemical changes in the rats’ brain and attenuated the levels of neuroinflammation (TNFα). Anti-HD effects of Qu are shown in Fig. (**2C**).

### 3.4 Amyotrophic Lateral Sclerosis (ALS)

ALS, also called Lou Gehrig’s disease, is a progressive neurodegenerative disease involving degeneration of upper and lower motor neurons. The degeneration of upper motor neurons leads to muscle stiffness and spasticity, whereas degeneration of lower motor neurons leads to spontaneous muscle twitching (fasciculation) and excessive electrical dysfunction. Symptoms of ALS include cramping, problem with coordination, muscle spasms, muscle weakness, stiff muscle, fatigue or feeling faint, difficulty in speaking, vocal cord spasm, innervation of the eye, and sphincter muscles [78, 79].

Qu has been reported to be effective in the treatment of ALS by attenuating several anti-ALS biomarkers such as SOD1, MDA and ROS [80]. It has also been reported for its anti- neuroinflammatory effects and attenuating the levels of NF-κB, TNF-α, IL-1β, IL-6, and IL-8 [81]. To date, only two studies have been reported for the effect of Qu specifically in ALS; however, several other studies have been reported where Qu showed inhibitory effects against potential ALS biomarkers. In one of the studies, Lazo-Gomez and co-workers reported the effects of Qu against α-amino-3-hydroxy-5-methyl-4-isoxazole propionic acid (AMPA) induced ALS in rats. AMPA leads to degeneration of spinal motor neurons and chronic excitotoxicity, which further causes neuronal apoptosis. AMPA also enhanced the level of sirtuin (SIRT1). Qu inhibited SIRT1 and reduced the effects of AMPA, which further resulted in the prevention of degeneration of motor neurons [82]. Another study showed the effects of two bioactive flavonoids, Qu and baicalein, which showed anti-amyloidogenic effects and attenuated cytotoxicity of SOD1 *in silico* study and *in vitro* MTT assay [83]. Effects of Qu against ALS are represented in Fig. (**2D**).

## 4 CLINICAL TRIALS USING QU

Many completed and ongoing clinical trials have been reported on Qu for the treatment of numerous diseases such as diabetes, hyperuricemia, gastroesophageal reflux disease, hypertension, inflammatory disease, and coronavirus disease (COVID-19). Till now, there are limited clinical trials conducted on Qu for the treatment of NDs. A possible cause for this could be a lack of comprehensive understanding of how Qu acts in the treatment of NDs. The completed and ongoing clinical trials have been summarized and listed in Table **1** [84].

## 5 CHALLENGES ASSOCIATED WITH THE DELIVERY OF QU IN CNS

Even though Qu possesses multiple pharmacological benefits, there are some major challenges associated with its delivery due to its Physico-chemical properties such as poor aqueous solubility, poor permeation, low systemic availability and extensive first-pass metabolism, and poor lipid solubility [85-87]. To overcome these challenges, various Qu-based NDDS have been formulated that have shown positive results [88]. Incorporating Qu in nano-formulations changes the physical nature of the drug that leads to improved dissolution rate, bioavailability, and pharmacological activity with reduced dose and protection from gastrointestinal (GI) degradation in comparison to the raw drug such as Qu [86, 89, 90]. Various NDDS have been reported so far in oral delivery of Qu, and the mechanism of NDDS transport to the brain is explained in Figs. (**3A**, **B**). In one of the studies, Dian *et al.* reported that Qu-loaded polymeric micelles exhibited 286.1 percent relative bioavailability which was 2.8 folds higher than that of raw Qu [89]. About 1.44 folds increase in the maximum concentration (Cmax) of Qu was observed in the nano-formulation. In another study, Wang *et al.* reported Qu-loaded self-double nano emulsifying drug delivery system (SNEDDS), showing 5.2 folds higher relative bioavailability than raw Qu suspension. They also reported that Qu SNEDDS enhanced the area under curved (AUC) (0-∞) of Qu by 5.2 folds and Cmax by 4.16 folds as compared to raw Qu suspension [91]. Various NDDS proposed so far and their applications in treating NDDS are discussed in Section 6.

## 6 QU-LOADED NDDS FOR THE TREATMENT OF NDS

### 6.1 Exosomes

**Fig. (4):**
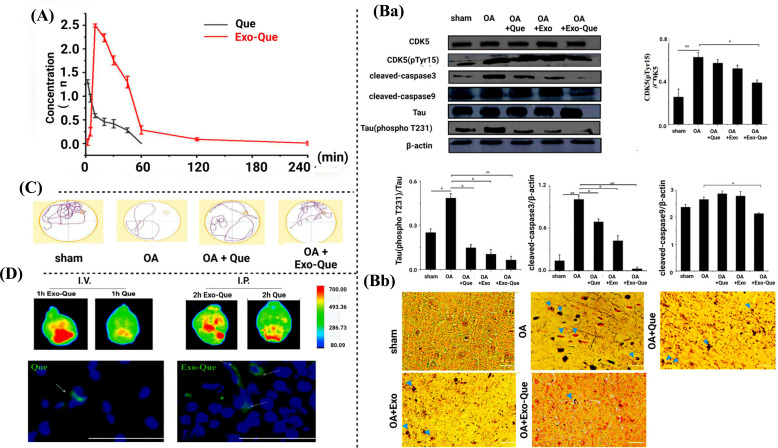
Anti-AD effects of Qu-loaded exosome [92]. (**A**) Bioavailability study (**Ba**) Effects of Qu-loaded exosomes in CDK5(pTyr15)/CDK5, Tau (phospho T231)/Tau, cleaved-caspase3/β-actin and cleaved-caspase9/β-actin. (**Bb**) The image of NFT (blue arrows) on the brain section. (**C**) Behavioural parameters in MWM test. (**D**) Representative fluorescence images of C57BL/6 mice brains treated with Qu and Exo-Qu *via* I.V. injection and I.P. injection.

Exosomes are nano-sized vesicles secreted by living cells. They hold a promising potential as a drug delivery carrier for transporting drugs to a specific site or organ. Compared with other inorganic and organic carriers, exosomes possess many advantages such as good compatibility, innate stability, low immunogenicity, and high transmission efficiency [92]. In one of the studies, Qi *et al.* studied the neuroprotective effects of Qu-loaded exosomes in okadaic acid-induced AD in mice. The study revealed that Qu-loaded exosomes enhanced cognitive dysfunction and attenuated neurofibrillary tangles for the treatment of AD. The exosomes also exhibited improved brain targeting and bioavailability of Qu in comparison to its raw form. The pharmacokinetic study showed that exosomes increased the bioavailability of Qu by 7.45 folds (Fig. **4A**). To evaluate the anti-AD effects of Qu-loaded exosomes, various biochemical and behavioural parameters were evaluated. Qu-loaded exosomes inhibited over-phosphorylation of tau protein induced by okadaic acid. Okadaic acid enhanced the level of NFT in all the treatment groups except normal control. It also upregulated the levels of cleaved caspase 9 and cleaved caspase 3. Qu-loaded exosome minimized the production of NFT and reduced the phosphorylation of CDK5 and tau. Moreover, the expressions of cleaved caspase 9 and cleaved caspase 3 in the Qu-loaded exosomes treated groups were significantly downregulated when compared with the groups receiving raw Qu (Fig. **4B**). Qu exosomes treated groups showed shorter escape latency, higher target quadrant percentage, and an increased number of crossing in the Morris water maze (MWM) test (Fig. **4C**). In another study, intravenous injection (I.V.) and intraperitoneal injection (I.P.) of Qu exosome to the brain of C57BL/6 mice reduced the apoptosis pathways compared to Qu alone. This was observed through fluorescence imaging (Fig. **4D**).

### 6.2 Nanoemulsion

Nanoemulsion is a mixture of aqueous and oily phases stabilized by an emulsifying agent (surface active agent). It is a kinetically stable system where Brownian motion effects surpass the gravitational forces, resulting in greater tolerance to droplet aggregation than conventional emulsified systems [93, 94]. The average particle size of the nanoemulsion is 20 to 500 nm. Nanoemulsions have been reported to possess high drug loading capacity with better stability. In addition, it is non-toxic in nature [95]. It enhances the drug potency when administrated through oral, topical, nasal as well as parenteral routes [96].

### 6.3 Self-Nanoemulsifying Drug Delivery System (SNEDDS)

SNEDDS is the anhydrous and homogeneous mixture of specific oil, surfactant, and co-surfactant; when orally administered, it gets mixed with the gastric fluids and forms nanoemulsion in the stomach. The hydrophobic drug remains in the solution form at this stage and gets easily absorbed [97].

SNEDDS offers advantages as it increases the solubilization of the drug. It also inhibits the P-glycoprotein-mediated drug efflux and promotes the lymphatic transport of the drug [98].

In a study, Ahmad *et al.* reported that Qu-loaded in SNEDDS enhanced the bioavailability of the drug for the treatment of cerebral ischemia [99]. The Qu-loaded SNEDDS had a droplet size and zeta potential of 94.63±3.17 nm and -17.91±1.02 mV, respectively. The SNEDDS was developed for *in situ* formation of oil-in-water nanoemulsions [99].

### 6.4 Solid Lipid Nanoparticles

Solid lipid nanoparticles (SLNs) are a mixture of solid and/or lipid, surfactant, and co-surfactant loaded with bioactive substances and/or drugs. Many studies have been conducted on SLNs due to their nano size range, lipid nanoparticles based improved solubility, bioavailability, and the possibility of developing new therapeutics [100]. In addition, SLNs have been reported to possess higher drug loading and stability [100].

Rishitha and co-workers developed Qu-loaded solid lipid nanoparticles (SLNs) for the effective treatment of AD. In this study, pentylenetetrazole (PTZ) induced female zebrafish model was used to understand the inhibitory potential of Qu-loaded in SLNs. To evaluate the inhibitory effect, various behavioural parameters such as light and dark chamber test, three horizontal compartment tests, and estimations of partition preference were performed. Some biochemical tests such as GSH, TBARS, and AChE were also conducted, wherein the results of raw Qu, Qu SLNs, and donepezil treated groups showed significant (*p*<0.05) upregulation of GSH and downregulation of TBARS and AChE levels. The light and dark chamber test was evaluated through time spent in the light chamber (TSLC) and the number of entries to the dark chamber (NEDC) by the zebrafish, wherein the results indicated lesser TSLC and higher NEDC. The partition preference test was evaluated through percentage entry to the target chamber (% ETC), and time spent in the target chamber (TSTC); three horizontal compartment test was evaluated through time spent in the upper segment (TSUS) and time spent in the lower segment (TSLS). The PTZ treated groups showed lesser values of TSTC, % ETC and TSUS and higher values of TSLS, indicating PTZ induced memory dysfunction. The raw Qu, Qu SLNs, and donepezil (standard drug) treated groups showed (p > 0.05) a significant increase in the values of TSTC, %ETC and TSUS and a decrease in values of TSLS in a dose-dependent manner, indicating a reversal in memory dysfunction induced by PTZ in zebrafish.

### 6.5 Metallic Nanoparticles

Various metals such as gold, silver, platinum, and iron have been used for the preparation of metallic nanoparticles. These nanoparticles exhibit particles size ranging from 1 to 100 nm [101]. Metal nanoparticles exhibit various shapes like spherical, nanoshells, rod, hollow, diamond, *etc.* In one of the studies, Liu *et al.* developed Qu-loaded gold-palladium (AuPd) nanoparticles and analyzed their effect against Aβ aggregation and mHtt genes. The results indicated that Qu-loaded AuPd nanoparticles exhibited significant neuroprotective effects in AD in a concentration dependant manner. Concave cubic shaped Qu polysorbate 80 (P-80) stabilized AuPd core-shell structured nanoparticles were reported to cross BBB by endocytosis. Owing to their lipidic nature and nano size, Qu nanoparticles activated autophagy and minimized the Aβ aggregation in neuronal cells, which is the leading cause of the progression of AD. These Qu nanoparticles also showed neuroprotective effects by attenuating Aβ induced cytotoxicity. Cell viability, caspase3 activities, and LDH release were also evaluated. Qu nanoparticles at the dose of 5 and 10 μg/mL showed a higher inhibitory response as compared to raw Qu in SH-SY5Y cells (*p < 0.05 and **p < 0.01). The histopathological image revealed that Qu AuPd nanoparticles did not show any toxicity in the cortex, hippocampus, and thalamus parts of the mice brain in the hematoxylin and eosin staining assay as compared to the control group [102].

Liu and co-workers reported the neuroprotective effects of Qu-loaded to sulphur nanoparticles in combination with microbubbles and focused ultrasound, which enhanced the cognitive function and attenuated the brain cell injury associated with AD [103]. The study included biochemical parameters, behavioural as well as histopathological studies. In the biochemical evaluation, Qu sulphur nanoparticles were tested against ER stress in SH-SY5Y cells. Qu sulphur nanoparticles inhibited the level of lipopolysaccharide in BV-2 microglial cells. The anti-inflammatory activity of formulation was evaluated by various assays such as nitric oxide (NO), prostaglandins (PGE2), TNF-α, and IL-1β, wherein Qu sulphur nanoparticles produce anti-inflammatory activity by suppressing those inflammatory mediators compared with raw Qu and raw sulphur nanoparticles. In another cell line study, Qu sulphur nanoparticles have shown a good response against the accumulation of Aβ and thapsigargin (TG) induced ER stress and neuroinflammation in SH-SY5Y cells compared to Qu sulphur nanoparticles given individually.

### 6.6 Polymeric Micelles

Polymeric micelles are unique nanocarriers that entail a hydrophilic shell and hydrophobic core. Hydrophobic bioactive substances are loaded in the middle of the polymeric micelles. These are potential drug delivery systems that have been reported to enhance the solubility, bioavailability, and biocompatibility of the various drugs [104-106]. They offer significant drug loading and provide controlled release at the target sites [107, 108].

**Fig. (5):**
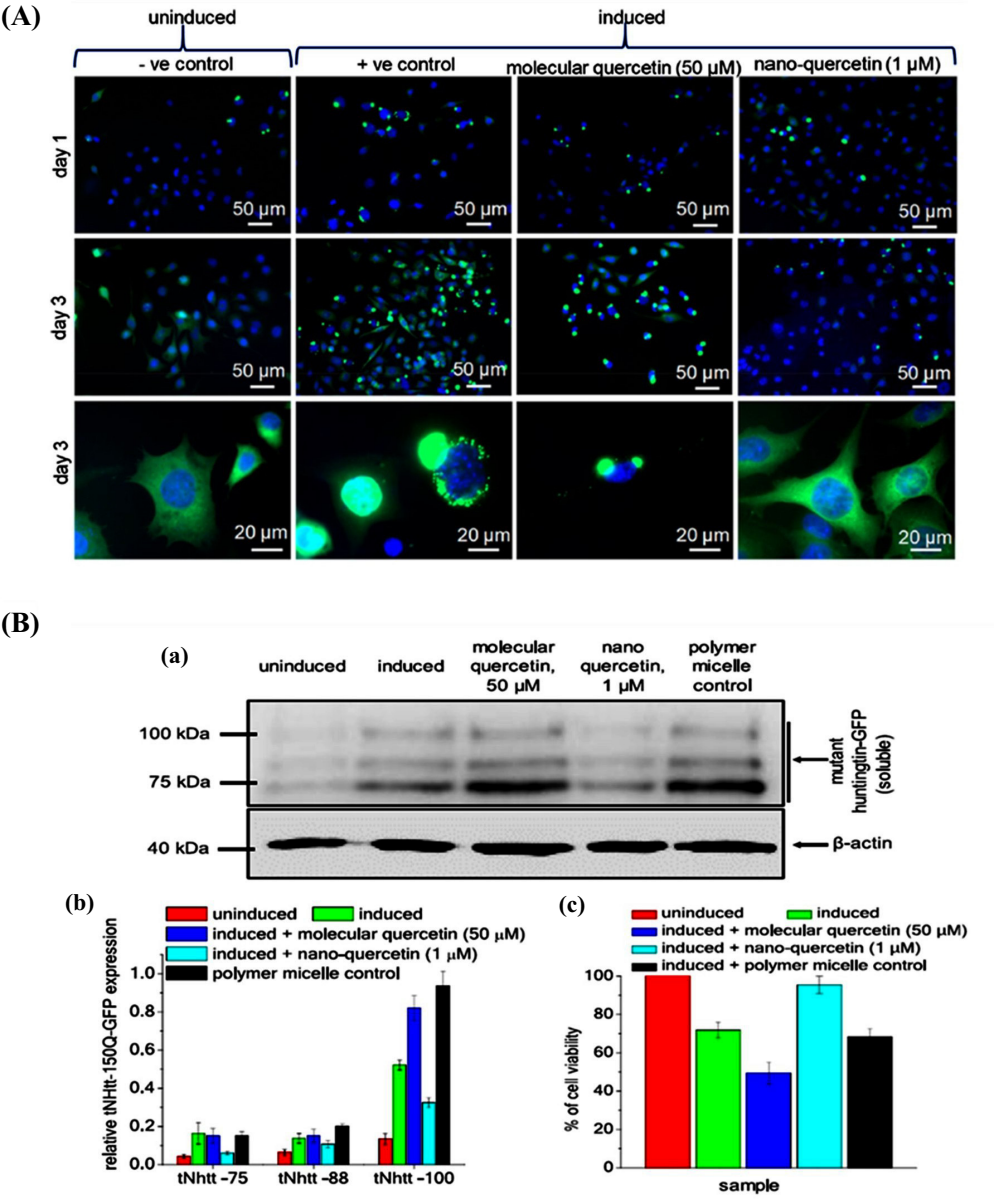
Qu nanoparticles minimize symptoms of AD in cell lines and *in vivo* studies [90]. [Copyright (2019) American Chemical Society]. (**A**) Three days observation studies in which observed effects of Qu nanoparticle, polyglutamate aggregation, and reduction in cytotoxicity. (**B**) Immunoblot assay.

Debnath *et al.* reported the neuroprotective effects of Qu-loaded polyaspartic acid-based polymeric micelles (Qu-PAPMs) for the treatment of AD and HD. Its efficacy was tested *in vitro* on ponasterone-induced HD150Q cells [90]. The neuroprotective effects were compared with the drug alone. The study was conducted for three days. The parameters tested were an aggregation of mHtt in HD150Q cells. The results indicated that Qu-PAPMs inhibited aggregation of mHtt on the first day itself, whereas raw Qu was unable to suppress the aggregation. In another study, the authors reported the neuroprotective effects of Qu-PAPMN on truncated mutant N-terminal huntingtin (tNhtt) aggregation (polyglutamine) inside the cell. Immunoblot assay and MTT assay were carried out using HD150Q cells (Fig. **5A**). In the immunoblot assay, a green fluorescent protein antibody was used as an inducing agent in HD150Q cells. Qu polyaspartic acid-based polymeric micelles and raw Qu were incubated for 72 h with various groups wherein Qu polyaspartic acid-based polymeric micelles inhibited aggregation of terminal Huntingtin (tNhtt) at a dose of 1 μM. The results showed better activity compared to raw Qu at a dose of 50 µM. Qu polyaspartic acid-based polymer micelles were separated to exhibit 95% survival of the cells compared to raw Qu (49%) (Fig. **5B**).

In another study, Dian *et al.* developed soluplus polymeric micelles loaded with Qu, which enhanced its oral bioavailability. The novel formulation was prepared by the modified film dispersion method, where the mean particle size was obtained as 79.00 ± 2.24 nm for the optimized batch. The study revealed 2.86 folds enhanced oral bioavailability of Qu-loaded in soluplus polymeric micelles [109].

### 6.7 Polymeric Nanoparticles

Polymeric nanoparticles have gained substantial attention in recent years owing to the unique features they possess due to their small particle size [110]. The advantages of polymeric nanoparticles as drug carriers include their potential for controlled release, their capacity to shield drugs and other biologically active molecules from the environment, and their ability to increase the bioavailability and therapeutic index [111, 112].

Zein nanoparticles loaded with Qu were found to enhance the bioavailability and produced neuroprotective effects against AD in the mice model [113]. The drug-loaded nanoparticles displayed a zeta potential, polydispersity index, and mean particle size of –43.0 ± 1.2 mV, 0.24, and 260 ± 8 nm, respectively. The study included various behavioural and biochemical parameters. In a behavioural study, various parameters such as open field test, rotarod test, and MWM test were performed. In the MWM test, improvement in cognitive function was observed as their escape latency (on days 4, 6, and 7) was significantly reduced compared to the positive control group. No significant differences were observed between the positive control group, and the raw Qu treated group.

**Fig. (6):**
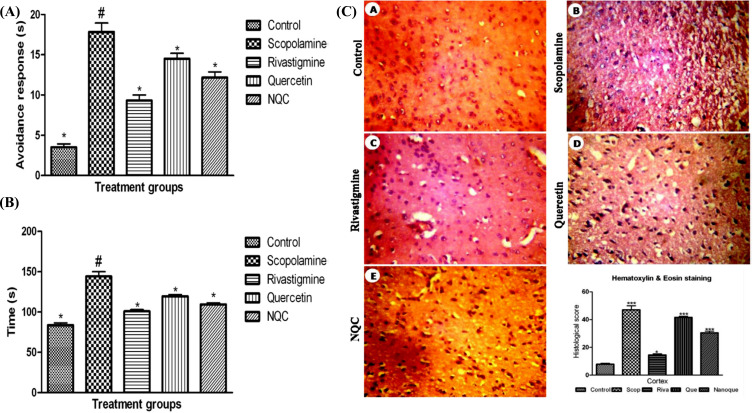
Effect of Qu nanoparticles in scopolamine-induced spatial memory deficit for the treatment of AD [114]. (**A**) Effect of raw Qu and Qu nanoparticles on avoidance test against scopolamine-induced cognitive dysfunction and mental confusion in rat, (**B**) Effect of Qu and Qu nanoparticles on rectangular maze test for analyzing cognitive function in rat, (**C**) Histopathology images indicating neuroprotective effects in the normal, control, raw Qu, Qu nanoparticles and standard drug.

Palle *et al.* conducted Qu nanoparticles-based behavioural studies in rats [114]. Qu nanoparticles showed neuroprotective effects against scopolamine-induced cognitive dysfunction and confusion. Cognitive ability was evaluated by avoidance test and rectangular maze test. Scopolamine at a dose of (20 mg/kg) was introduced i.p. to rats who received raw Qu (30 mg/kg, p.o.), Qu nanoparticle (30 mg/kg, p.o.), and rivastigmine (2 mg/kg, i.p.) as standard control. The results of the avoidance test and rectangular maze test showed a significant reduction in transfer latency and an increase in cognitive ability in rats treated with Qu nanoparticles as compared to rats receiving raw Qu (Fig. **6A** and **B**). A non-significant difference was observed between Qu nanoparticles and rivastigmine. In addition, histopathological studies were carried out in eosin, and hematoxylin-stained sections of the brain, wherein the control group showed abnormal cellular morphology and gliosis effects. The Qu nanoparticles were able to reduce the abnormality and cellular morphology similar to rivastigmine (Fig. **6C**) [114].

The Qu nanoparticles were developed by Han and co-workers for the treatment of AD in SH-SY5Y neuroblastoma cells. The developed Qu nanoparticles exhibited an average particle size of 50 nm. Qu nanoparticles helped in the reduction of oxidative stress induced by Aβ, Aβ mediated cytotoxicity, and Aβ42 assembly. The developed Qu nanoparticle decreased the level of ROS that was induced by Aβ42 and H_2_O_2_. Raw Qu also reduced the level of ROS, but Qu nanoparticle completely neutralized its level. These results were similar to the control group, which represented that the Qu nanoparticle exhibited a unique ROS scavenging activity [115].

Ghaffari1 *et al.* reported various neuroprotective effects of Qu nanocrystals against 6-hydroxydopamine (6-OHDA) for the treatment of PD in male rats. Qu nanocrystals were prepared by the evaporative precipitation of the nanosuspension method. Raw Qu and Qu nanocrystals at the dose of 10 and 25 mg/kg enhanced cognitive function and attenuated the level of SOD, CAT, and total glutathione, and ameliorated the level of MDA in the hippocampus part of the brain in rats [116].

In another study, Rifaai *et al.* reported neuroprotective effects of Qu nanoparticles through performing various histopathological and ultrastructure studies using transmission electron microscopy in adult male SD rats [88]. In this study, rats were divided into four different groups. The first group was administrated vehicle of Qu nanoparticles. The second group received aluminium chloride (Al_2_Cl_3)_ (100 mg/kg) as an AD inducing agent. The third group received Qu nanoparticles (prophylaxis group) with coadministration of Al_2_Cl_3_. The fourth group received Qu nanoparticles (treatment groups) after 42 days of administration of Al_2_Cl_3_. The prophylaxis group and treated group of Qu nanoparticles inhibited the production of neutrophil and granular cells, but the Qu nanoparticle treated group (group IV) has shown 1.29 folds better effects as compared to prophylaxis groups (group III). Qu nanoparticles treated group (group IV) inhibited 0.9 folds of tyrosine hydroxylase activities as compared to Qu nanoparticles prophylaxis group (group III). Qu nanoparticles treated group (group IV) showed significantly (p ≤ 0.05) better response as compared to Qu nanoparticles prophylaxis group and downregulated the formation of neurofibrillary tangle, and Aβ by6.44 folds and 2.1 folds, respectively. Ultrastructural changes were observed in electron micrographs in the AD induced group (group II), whereas treatment with Qu nanoparticles inhibited astrocytes, and microglia and prevented neuroinflammation.

## CONCLUSION

Neurodegenerative diseases become chronic with age. Various molecular mechanisms of NDs that are responsible for progression include oxidative stress, neuroinflammation, impaired DNA repair, mitochondrial dysfunction, and accumulation of abnormal protein into the brain; however, a potential treatment for the NDs is yet to be identified. Several synthetic drugs have shown neuroprotective effects, but they are associated with unitarget action and more side effects. To overcome this challenge, multitarget drug therapy is needed, which has higher potency with minimum side effects. Qu has been reported to exhibit multiple molecular pathways for the treatment of NDs. Qu-nanoparticles have been found to enhance potency, water-solubility, bioavailability, and BBB permeability. Colloid-based lipid nanoparticles of Qu enhanced the quality, safety, and stability of the formulation. To sum up, Qu nanoparticles hold a great potential in increasing brain tissue regeneration, reducing oxidative stress, reversing mitochondrial dysfunction, decreasing neuroinflammation, stopping abnormal protein formation, and displaying anti-aging effects.

Despite these facts, the clinical translation and commercialization of Qu-nanoparticles are challenging due to certain bottlenecks that include large-scale manufacturing, biological challenges, biocompatibility and safety, intellectual property (IP), government regulations, and overall cost-effectiveness in comparison to currently available therapies. A correlation between disease pathology and heterogeneity related to NDs among humans should be made. Furthermore, the physicochemical properties of Qu-nanoparticles should also be a prime focus for overcoming biological barriers to achieve better targeting of drugs to diseased tissue as well as a reduction in their accumulation in non-specific organs. Unfortunately, less attention during research has been given to these aspects to comprehensively understand the correlations between Qu-nanoparticles behavior and patient biology. This could be one of the major reasons for the failure observed during the translation of promising nanoparticles in clinical trials. These biological barriers could be a significant deterrent for pharmaceutical industries for investing in Qu-nanomedicines. To overcome this challenge, a comprehensive evaluation of preclinical data is required in terms of therapeutic efficacy, safety, biodistribution, and pharmacokinetics in appropriate animal models of NDs. To get reproducible results, the trials must be done using different animal models of NDs rather than relying on a single animal model because these models reflect only a narrow correlation with human physiology. Considering the aforementioned challenges associated with nanoparticle development and overcoming them will help in better clinical translation of Qu- nanoparticles for their biomedical application in NDs.

## Figures and Tables

**Fig. (3):**
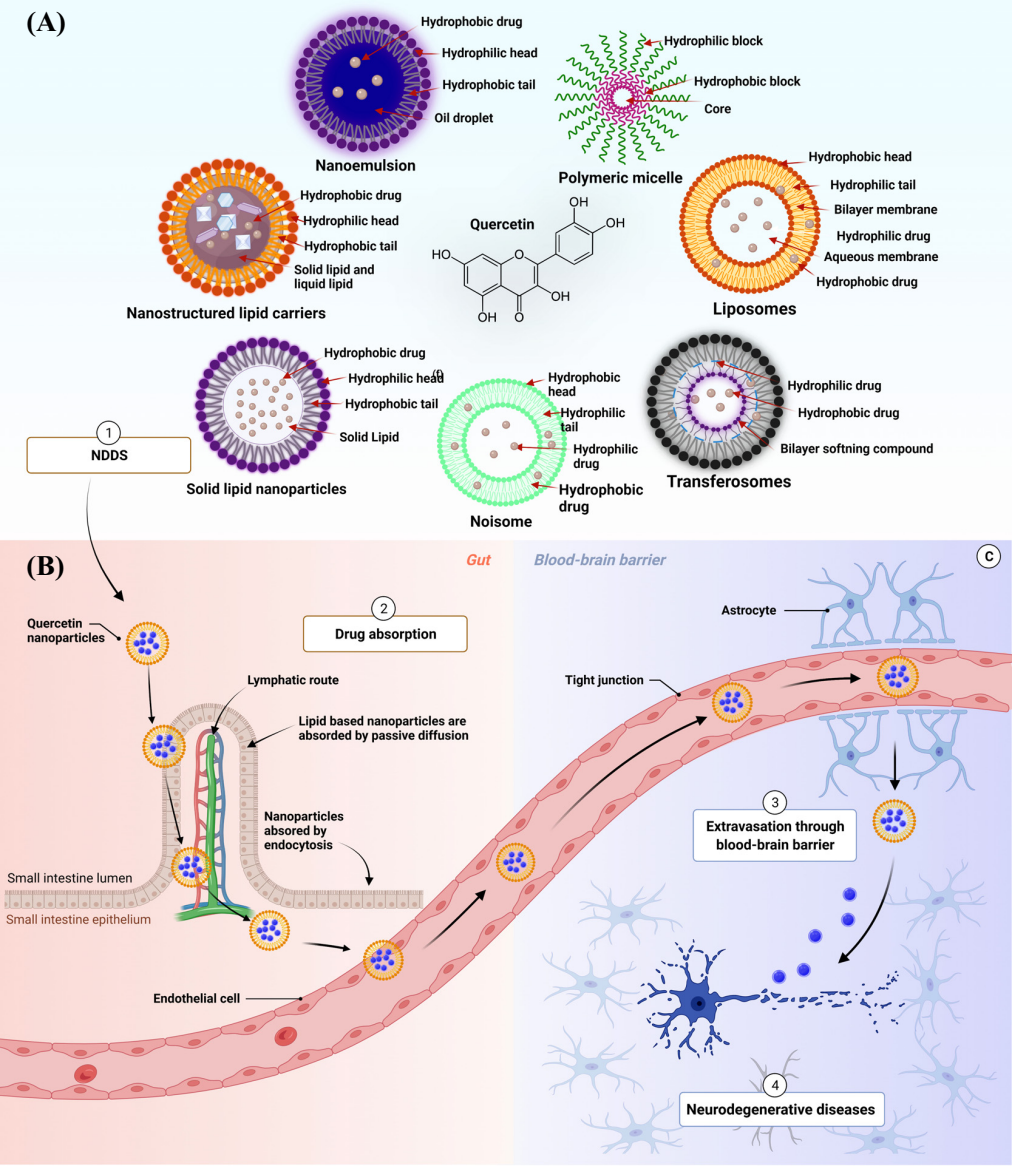
Figure depicting (**A**) various nanoparticles prepared for Qu and (**B**) mechanism involved in brain targeting of Qu NDDS upon oral administration. Figure depicting (**A**) various nanoparticles prepared for Qu and (**B**) mechanism involved in brain targeting of Qu NDDS upon oral administration. 1. Various NDDS such as NEs, nanoliposome, SLNs, NLCs, polymeric nanoparticles, transferosomes, and niosome; (**B**) 2. The Qu-loaded NDDS get absorbed by passive diffusion and endocytosis and reach into systemic circulation; 3 These NDDS cross BBB because of their nanometer size.

**Table 1:** Summary of the completed and ongoing clinical trials of Qu [84].

**Completed Clinical Trials**							
**Disease**		**Intervention**	**Sample Size**	**Phase**	**Study Start Month**	**Estimated Study Completion Date**	**NCT Number**
COVID-19		Qu Phytosome	NA	142	January 2021	August 2021	NCT04861298
COVID-19		Qu Phytosome	152	3	September 2020	April 2021	NCT04578158
COVID-19		Qu	NA	447	March 2020	August 2020	NCT04377789
Hyperuricemia		Uricemin, placebo	4	116	October 2018	December 2019	NCT04161872
Gastroesophageal Reflux Disease		Qu	26	1	August 2014	June 2016	NCT02226484
Chronic obstructive pulmonary disease		Qu and sugar chew	9	1	February 2014	October 2015	NCT01708278
Adenocarcinoma of the Prostate Recurrent Prostate Cancer Stage I, IIA, IIB, III, IV Prostate Cancer		Green tea extract, Qu, placebo	1	32	January 2014	June 2021	NCT01912820
Healthy Overweight Obese		Onion peel extract	4	62	April 2013	December 2013	NCT02180022
Hyperuricemia, gout, kidney Calculi, diabetes cardiovascular disease		Qu	1	22	February 2013	November 2014	NCT01881919
Hypercoagulable States		Isoquercetin and Qu	38	Early Phase 1	November 2012	October 2020	NCT01722669
Hypertension Endothelial Dysfunction		Epicatechin, Qu and Placebo	NA	38	September 2012	March 2013	NCT01691404
Mild to Moderate AD		Etanercept, Bio-Curcumin, Omega-3, Qu, Resveratrol	12	1	February 2010	May 2016	NCT01716637
Chronic Hepatitis C		Qu	1	34	July 2011	June 2014	NCT01438320
Obesity, Diabetes		Qu and placebo	2	24	April, 2010	August 2021	NCT00065676
**Ongoing Clinical Trials**							
AD, Early Onset Mild Cognitive Impairmen	Dasatinib, Qu and Placebo Capsules		48	2	December 2021	January 2032	NCT04685590
Prostate Adenocarcinoma	Qu, Bromelain, Rye Flower Pollen & Papain		2	140	August 2021	April 2025	NCT04252625
Severe Acute Respiratory Syndrome	Qu and placebo		200	Early Phase 1	June 2021	August 2021	NCT04853199
Coronary Artery Disease	Qu, placebo		2	100	June 2021	June 2022	NCT04907253
Aging	Dasatinib plus Qu		25	2	December 2020	June 2022	NCT04946383
COVID-19	Qu, bromelain, Zinc, vitamin C		4	60	June 2020	July 2020	NCT04468139
AD	Dasatinib and Qu		5	1,2	February 2020	August 2023	NCT04063124
COPD	Qu		1 and 2	15	October 2019	July 2021	NCT03989271
Coronary Artery Disease Progression	Qu		3	60	August 2019	June 2022	NCT03943459
Fanconi anaemia squamous cell carcinoma	Qu		2	55	May 2018	September 2023	NCT03476330

**Abbreviation:** R; Randomized, COVID-19; **Note:** coronavirus disease of 2019 https://clinicaltrials.gov/ct2/results?cond=quercetin&term=&cntry=&state=&city=&dist= [Accessed 16. Feb 20^22^]

## LIST OF ABBREVIATIONS

Aβ: Amyloid β
ACh: Acetylcholine
AD: Alzheimer Disease
ALS: Amyotrophic Lateral Sclerosis
ApoE: Apolipoprotein E
ATXN2: Ataxin2
Au-Pd: Gold-Palladium
BBB: Blood Brain Barrier
CAG: Cytosine-Adenine-Guanine
CAT: Catalase
COVID-19: Coronavirus Disease
CNS: Central Nervous System
CUS: Chronic Unpredicted Stress
Cu/ZnSOD: Zinc Containing Superoxide Dismutase
ER: Endoplasmic Reticulum
FUS: Fused in Sarcoma
GABA: Gamma-Aminobutyric Acid
GFAP: Glial Fibrillary Acidic Protein
GPX: Glutathione Peroxidase
H_2_O_2_: Hydrogen Peroxide
HD: Huntington Disease
HNE: Hydroxynonenal
IL: Interleukin
I.V.: Intravenous Injection
LB: Lewy Body
MAPK: Mitogen-Activated Protein Kinase
MDA: Malondialdehyde
mHTT: Mutation Huntingtin
MPP: 1-Methyl-4-phenyl Pyridinium
MWM: Morris Water Maze
NADPH: Nicotinamide Adenine Dinucleotide
ND: Neurodegenerative Disease
NDDS: Novel Drug Delivery System
NEs: Nanoemulsions
NF-κB: Nuclear Factor Kappa a Light Chain Enhancer of Activated B Cells
NFT: Neurofibrillary Tangle
NLCs: Nanostructured Lipid Carriers
O_2_•: Nitric Oxide
OH•: Hydroxyl Radicals
OPTN: Optineurin
PD: Parkinson’s Disease
PNS: Peripheral Nervous Systems
Qu: Quercetin
SIRT1: Sirtuin
ROS: Reactive Oxygen Species
RNS: Reactive Nitrogen Species
SD: Sprague Dawley
LNs: Solid Lipid Nanoparticles
SNEDDS: Self-Nanoemulsifying Drug Delivery System
SOD: Superoxide Dismutase
SQSTM1: Sequestosome 1
TBK1: TANK-binding Kinase 1
THP-43: Tammhorsfall Glycoprotein-43
ThT: Thioflavin T
TNF: Tumor Necrosis Factor
UV: Ultraviolet
